# Thrown off track? Adjustments of Asian business to shock events

**DOI:** 10.1057/s41291-021-00158-y

**Published:** 2021-06-11

**Authors:** Sierk Horn, Tomoki Sekiguchi, Matthias Weiss

**Affiliations:** 1 Hochschule München, Munich University of Applied Sciences, Am Stadtpark 20, 81243 Munich, Germany; 2 Graduate School of Management, Kyoto University, Yoshida-Honmachi, Sakyo-ku, Kyoto, 606-8501 Japan; 3 Center for Entrepreneurship, Innovation, and Transformation, Ruhr-University Bochum, Suttner-Nobel-Allee 4, 44803 Bochum, Germany

**Keywords:** Shock, Uncertainty, Resilience, Asian business, Cultural differences

## Abstract

The need for a better understanding of radical uncertainty might have never been greater. Ill-preparedness for natural hazards, a resurgence of serious public health concerns or illusions of control over unruly technology question the extent to which we can ‘really’ shape the world around us. Human-made crises, too, test how we routinely do things. We ask how organisations and actors within them prepare for a collapse of meaning and practise radical uncertainty. Given the breadth and depth of the region’s energy (and, as some would argue, turbulences), Asia provides a fitting context for exploring accommodation to and learning from low-probability, high-impact incidents. By reviewing the business and management research on shocks in Asia, we find that there is a strong human side to dealing with the unknown. We argue that what organisations and actors within them do prior, during and after a shock event is substantially contingent upon cultural environments. To elaborate, we discuss the role of the uncertainty avoidance dimension of national culture in dealing with shock events. We further combine this dimension with the universalism-particularism dimension to discuss future research directions. Our exploration of resultant differences in preparedness, resourcefulness and learning offers a more rounded inquiry into how Asian business actors deal with shocks.

## Introduction

The sheer speed and global scope of international business operations today are astonishing. An interconnected world has, however, fundamentally altered the rules of doing business (Mack et al., 2015). The Covid-19 pandemic quite plainly shows Asia’s tremendous impact on many firms’ growth (and even survival) in the (rest of the) world. While business in the Asia–Pacific rebounds, continued and complex lockdown measures, for instance in Europe, mean that industries struggle serving clients across Asia and beyond. Of course, international activities foster vulnerabilities (Liesch et al., 2011)—such as how to deal with country risks, enter overseas markets, manage linguistic or cultural differences in day-to-day business and so forth.

Firms expanding to Asia, too, are no strangers to volatility, uncertainty, complexity and ambiguity (VUCA for short, Schoemaker et al., 2018): Proxy involvement in human rights violations (e.g. Volkswagen in Urumqi), insufficient preparation for political interventions (e.g. application of the Chinese social credit system to companies), worries about the well-being of employees (e.g. cases of infection with coronavirus) or the flare-up of historical conflicts with unpredictable economic consequences (e.g. anti-Japanese demonstrations in China, 2015) test abilities to tilt unstable environments in one’s favour. But it does not stop with economic or political shocks that throw decision-makers off track; onsite projects often run differently than expected, turbulences occur at ever shorter intervals and at some point bring about major changes.

The fragile alliance between Nissan, Renault and Mitsubishi (Lewis et al. 2019) or the Goldman Sachs corruption scandal in Malaysia (Makortoff, 2019) show how system pressure and suppressed knowledge can trigger a chain of events that lead to unpredictable results. In spite of such risks and imponderables, it is always easier to hold on to cherished practices, even abroad! No wonder decision-makers are hardly prepared to innovate when expanding to Asia, much less so during disruptive episodes (Lin, 2000). In short, question remain what organisations and actors within them can do about true Knightian uncertainty when risk is impossible to calculate, and decision making is no longer a neat, step-by-step process (Liu & Froese, 2020).

Our knowledge of business and management in Asia is relatively advanced. Yet, there has been a relative neglect of research on how organisations operate in non-routine landscapes and “practise” latent dangers (Müller-Seitz, 2014). This shortage of knowledge is in sharp contrast to a rash and random world that no longer allows for predictions with any degree of certainty, i.e. it will only become clear in the future what consequences past decisions have had. Radical uncertainty (as a consequence of events falling outside the horizon of expectation; Tsoukas, 1996) distorts efforts to understand the future and, by extension, response planning (Bennett & Lemoine, 2014). In the worst case, we cannot even anticipate threats (Buckley & Casson, 2019). This article, and the special issue of Asian Business and Management^1^ it introduces, takes this challenging question heads on and focuses on adjustment processes to an international corporate environment, in which organisations must strengthen responsiveness and resourcefulness. Our purpose here is twofold: (i) we will review how organisations from Asia and actors within them deal with shock and (ii) based on this, we will explore how cultural antecedents might affect the way how they practise uncertainty.

We are at the dawn of an era of a more assertive Asia (Mahbubani, 2020). From China’s Belt and Road Initiative and political sabre-rattling (e.g. Hong Kong’s national security law) to the ‘quiet’ transformation towards the world largest trading bloc (the newly formed Regional Comprehensive Economic Partnership (RCEP) will cover a quarter of the world population and a third of the world’s economic output), the burgeoning middle class of South-East Asian economies and, by extension, vibrant digital cultures of the Asia Pacific (most notably in terms IP specialisation or unicorns) showcase the region’s economic, politic and social capacities. Arguably, these shifts change the engagement between Asian economies and between them and Europe. The way regions across the world handle the recent COVID-19 pandemic and its social and economic fallout put on display such recalibrations: Here the tumbling giants of Europe (with a to and fro of imposing and relaxing lockdown measures, political impasses and “muddling through”), there the quick “V-shaped” recovery from the economic slump across many Asian economies (Liu et al., 2020). In short, recognition of Asia as a growth motor for global business comes with a growing appreciation for actors from this region and together make a timely context to explore how organisations cope with hard-to-predict and disorientating events.

We define shocks as incidents that (i) happen suddenly and unexpectedly; (ii) rupture our trust in how we usually go about things; and (iii) bring about a shifting reality that causes controversy about routes to adjustment. Clearly, for organisations, it is impossible to prepare for all eventualities adequately (Chakrabarti, 2015). Even if we expect an incident, we cannot predict the experience (King, 2016). Because of their low probability, shocks usually fall outside well-practised frames for identifying, assessing and prioritising risks (Lampel et al., 2009). Yet, in our view, we ought to emphasise hard-to-predict events in management. Shocks are not only possible but common in a progressively destabilising world (Giddens, 1990; Liu & Froese, 2020), where sudden losses of meaning pose a considerable threat to the functioning of organisations (van der Vegt et al., 2015). Crucially, if change unfolds in an unorderly manner, organisations and actors within them need to give meaning to experiences that rain down on them (Weick, 1993). A crack in the shelter of cherished routines often whirls one’s sense of agency. We feel unprepared and at a loss how to go about a situation, precisely because we cannot project the future from the past (Bernstein & Bernstein, 1996).

Generally, we can systematise the manifold types of shocks into different categories of shocks, connected to different properties of these shocks. As a very basic distinction, there are two broader categories of shocks; on the one hand, shocks based on natural occurrences like natural disasters or infectious diseases. On the other hand, shocks following man-made actions and decisions like regulatory shocks, market shocks, or different kinds of organisational shocks. These two broader categories differ in the cause of the shock and the ability to influence this cause of the shock, which is not the case for the shocks based on natural occurrences. In contrast, people more generally and organisational actors, in particular, can influence, at least indirectly, the cause of man-made shocks. This has implications for both crisis management during the shock experience and the post-shock outcomes (Bundy et al., 2016; Liu & Froese, 2020). Being able to influence the cause of a shock allows taking actions to prevent such shocks in the future, while this is not possible for natural disasters or newly emerging and spreading diseases. For these latter cases, people in general and organisational actors, in particular, can only try to develop safeguards and processes that reduces the negative impact by such occurrences in the future, like building (more) earthquake-resistant infrastructure, developing templates for vaccines that can be adapted to new diseases, or improving emergency reactions. Hence, organisational post-shock strategies might vastly differ in what can be learned from a shock experience for these two categories. Learning from a nature-based shock experience is mostly confined to a strategy focussing on better forecasting and developing resilience. This means that organisations take care that they are able to detect such a shock as early as possible and that they and their members are negatively affected to the least possible extent and that another occurrence of the same shock will cause less harm in any respect (Linnenluecke, 2017).

The specific adaptions for this purpose, especially to develop better resilience, might take quite diverse forms, e.g. developing social, relational, or human resources (e.g. Gao & Ren, 2020; Yakob, 2020; Zhang et al., 2021a, 2021b), and is likely, as we elaborate below and Hoegl and Hartmann (2021) outline in their perspective article in this special issue, to be substantially contingent upon cultural aspects. Specifically, Hoegl and Hartmann (2020) argue that resilience is a key concept in explaining why some entities positively adopt or even emerge stronger (i.e. bouncing back or beyond), while others suffer from setbacks. Defining resilience as positive adaptation within the context of significant adversity, Hoegl and Hartmann point out that a significant setback can provide an opportunity for development and growth, whereby individuals, teams, or larger collectives become stronger and more capable than before. However, based on an explanation of the resilience process, Hoegl and Hartmann also point to three major challenges for resilience research that are needed to be tackled in order to provide evidence-based recommendations how this enhanced strength and capabilities from shocks or other adverse events can be realised. In particular, they mention the need to consider individual to collective resilience or cross-level influences, to take events seriously and to identify universal as well as culture-specific antecedents of resilience.

In addition to such a forecasting and resilience-focussed strategy, organisations may attempt to avoid or minimise shocks caused by human action. This approach, which goes with the proverbial saying that to the extent we can control the future, we don’t need to predict it, can involve exerting the direct or indirect influence of whatever kind on key legislators, decision-makers, or other key stakeholders. However, not only the strategies to learn from a shock and to prepare for similar shocks in the future might differ vastly. In a similar vein, organisations may take highly different approaches when it comes to responding to a shock. In this regard, the cause of the shock, whether man-made or based on natural occurrences, does not seem to play a major role for organisations. Rather, organisations need to find a way to regain their operability, competitiveness and profitability after the adverse impact they suffered from, which normally implies major changes and adaptions in what they do and how they do it. The nature of these changes and adaptions is also contingent upon cultural factors.

Under these circumstances, non-ergodic conditions, management needs practically relevant guidelines for shaping their organisations and business, given the new risk reality. Arguably, Asia’s corporate environment, which is highly diverse and can be extremely turbulent (Chan & Cui, 2016), provides an excellent sounding board to learn from competent responses, which is why we will review the Asian-based literature on shocks in the following section.

## Research on shocks in Asia

To review the rich body of business and management research on shocks in the Asian context, we built on the different categories of shocks as mentioned above. Table 1 provides specific examples for these shock categories and studies dealing with them and their consequences. In our review of this literature, we observed four interesting general patterns.

**Table 1:** Organisational research on shocks in the Asian context

Shock category	Natural occurrences		Human actions		
Shock type	Natural disasters	Epidemics/pandemics	Market shocks	Regulatory shocks	Organisational shocks
Examples	Earthquakes Floodings Tropical storms	Covid-19 SARS AIDS	Resource shortages Financial crises Price increases	Pollution regulation Tax/wage reforms Deregulation	Business re-organisation Corporate scandals Accidents
Exemplar sources	Kong et al. (2021) Olcott and Oliver (2014) Zhang et al. (2012)	Dai et al. (2021) Habel et al. (2020) Lee and Warner (2006)	Dieleman (2010) Marino et al. (2008) Ramirez et al. (2012) Wong (2021)	Huang et al. (2021) Zhang et al. (2021a, 2021b) Zheng and Wang (2020)	Corbet et al. (2021) He et al. (2016) Sun et al. (2019)

First, business and management research on shocks in Asia predominantly dealt with shocks based on human actions. Among these, much attention is directed to the consequences of regulatory shocks (Huang et al., 2021; Zhang et al., 2021a, 2021b; Zheng & Wang, 2020), market-based shortages or price increases of raw materials (Wong, 2021) and financial crises (Marino et al., 2008) to organisations and how they manage their business. This is noteworthy as Asian countries, and especially the prominent economic players like China, India and Japan, are prone to different large-scale natural disasters like tropical storms and earthquakes, given their geographical location. Hence, beyond the recent surge of research on the Covid-19 shock and its implications for businesses (e.g. Dai et al., 2021; Liu et al., 2020; Park et al., 2021), a larger share of research in this direction could have been expected. This might hint at a higher level of salience and immediacy of regulatory and market-based shocks in Asian countries and a higher volatility in this regard. Alternatively, it might also point to a stronger focus of Asian countries on those kinds of occurrences and problems that managers can influence to a larger extent.

A unique example of studying the coincidence of two different shocks or threats is offered by Meyer-Ohle (2021) in this special issue, who thus goes beyond previous work that used to focus on the consequences of one single shock. Through a case study, Meyer-Ohle (2021) analysed how convenience store (CVS) operators in Japan responded to the two interconnected ground societal challenges in Japan: the ageing population, a slow-moving and predictable threat and the Great Eastern Japan Earthquake of 2011, a sudden and unexpected shock. Meyer-Ohle found that although CVS operations displayed weaknesses in terms of disaster readiness, the CVS companies demonstrated disaster resilience in which individual leadership, initiative and sacrifice of franchise owners had been instrumental to overcoming the challenges. The events of the 2011 disaster also helped companies gaining acceptance in communities as essential infrastructure providers that can also address the ageing population. In short, Meyer-Ohle found that responding to and involvement with these grand challenges became an essential part of the evolving business models of CVS.

Second, a substantial share of this literature deals with the level of risk taken by companies and organisational actors in crisis management responses and connected to post-shock outcomes like learning and strategy changes (Chan & Cui, 2016). A higher level of risk in this connection might involve responding to the shocks with innovation in terms of technologies, products, or organisational processes in order to adapt to the shock experience (Park et al., 2021). Lower levels of risk are reflected in adaptations based on imitation or other non-innovative changes in business management like building up resource slack, investing in insurances, reduction of corporate spending, or even reducing innovativeness through more conservative product portfolios or organisational processes (Habel et al., 2020).

Ha (2020) provides insightful research on such low-risk strategies and on these strategies’ outcomes for learning and capability development. Situated in the non-routine environment of eco-innovation in South Korea, which involves a radical and systematic transition from profit-oriented business models to sustainable ones, Ha (2020) looks at the role of imitation and multinational enterprises (MNEs) as a potential referent for local firms for making decisions on eco-innovation. Ha seeks to answer the following question: Under what conditions can foreign MNEs be possible imitation targets among local firms in a host country? Using the dataset based on the South Korean Innovation Survey, Ha found that foreign MNEs affect local firms seeking social proof from successful peers. Social proof found in successful MNEs can address local firms’ anxiety about bounded information-processing capabilities. However, preferences for social proof in local firms can be weaker if a firm’s own past experience is sufficiently strong. Also, the results also indicate that imitation of foreign MNEs may not lead to learning and development of eco-innovation capabilities in local firms. Generally, in the Asian context, there does not seem to exist a predominating pattern of how companies or managers react to shocks, which might be (also) due to intra-Asian differences in national cultural value, as we elaborate below.

Third, while research on shocks in the Asian context features studies from many different Asian countries, the vast majority of studies originates from China, Japan, Taiwan, Singapore and India. Hence, there is a sharp contrast regarding our knowledge of national or cultural peculiarities in Asian countries and their connection to the management of shocks between a small number of well-researched countries and a large number of countries, from which we only have scant empirical evidence on the management and consequences of shocks. What is more, some of the more than 40 Asian countries even represent utterly uncharted territory in terms of the management and consequences of shocks for businesses. Therefore, more empirical research is clearly needed in many Asian countries to allow for more culture-specific recommendations on how to deal with shocks in these contexts and how these recommendations might differ from other (Asian) countries. This becomes even more apparent when considering the great diversity of Asia with regard to cultural aspects, religions, languages, geographical settings and historical origins of societies (Cauquelin et al., 2014).

Aman et al. (2021) take a valuable step into this direction by reporting a study from the Central Asian country of Kazakhstan, thereby eradicating this previously blank spot on the map of research on shocks in Asian businesses. Through an in-depth case study in the healthcare sector under the introduction of State-Guaranteed Health Benefits Packages (SGHBP) as a sudden and unforeseen shock on small and medium enterprises, Aman et al. investigated how the external shock of SGHBP changed the balance between the elements of an entrepreneurial ecosystem wherein migrant women entrepreneurs (MWE) are a focal actor. Looking at pre-shock, shock and post-shock phases in their case analysis, Aman et al. found that the MWE’s adaptation to the external shock took place in collaboration with ecosystem actors, and the government provided the resources and financial support to overcome the challenges. The opportunity generated by the external shock is exploited through the unique competitive advantage of each ecosystem’s actors, manifesting the complementarity among the actors. The aim of attaining the envisioned joint value proposition acts as a cohering tool among an entrepreneurial ecosystem’s elements. The complementarity and coherence among an entrepreneurial ecosystem’s elements co-create ecosystem resilience and generate additional opportunities that entrepreneurs might exploit. Similar to the CVS case by Meyer-Ohle (2021), Aman et al.’s case illustrates how the actors facing the unexpected shock attained the positive adaptation after the external shock.

Fourth, Asian organisations, like all organisations, vary by industry (sector), by experience (time) and by heritage (history, country of origin). It would, therefore, be futile to propose ‘one’ Asian way of adjusting to a VUCA world. Yet, overall, there seem to be far less compartmentalising, deductive, or context-insensitive principles guiding how Asian firms seek to practice uncertainty. Yes, Asian organisations have been shown to meet uncertainty with imitation strategies (Buckley et al., 2007; Horn & Cross, 2016), control mechanisms (Gong, 2003; Lee et al., 2014), or avoidance responses (Fukao & Wei, 2008; Pak & Park, 2004). That is, responses to uncertainty have much in common with those of their Western counterparts.

However, social and organisational culture and related value systems play a particularly strong role in buffering volatility. Japanese firms, for instance, have been characterised as knowledge-creating networks (Nonaka & Takeuchi, 1995) displaying a high commitment to incrementalism (Makino & Beamish, 1999) and, by extension, learning (Delios & Henisz, 2000). Korean firms, too, display experiential learning capabilities. In response to shock experiences surrounding the Finance crisis, their risk-averse behaviour is now complemented by more flexibility and preparedness to innovate (Park et al., 2006).

By contrast, and at first blush, Chinese approaches to practising uncertainty appear to come with more unconventional risk-taking behaviour. This, however, is accompanied by an understanding of interdependencies of actions, most notably relationship building (Opper et al., 2017; Quer et al., 2019) while carefully accumulating abroad experience (Liu et al., 2016). Extant literature, therefore, suggests flexibility, realism and pragmatism with which Asian organisations handle hard-to-predict and disorientating environments. Thus, openness to change and commitment to organisational learning seem to be prominent features of them developing resilience.

Taking together these patterns we observed in our literature review, it becomes clear that attitudes towards uncertainty and aspects of national culture are likely to play an important role with regard to companies’ and managers’ responses to shocks and these responses’ effectiveness in the respective national environments. Focussing on the showcase cultural dimension of uncertainty avoidance, given that it represents a cultural dimension closely connected to individuals’ risk-related behaviours, we discuss potential differences and commonalities between Asian countries in companies’ and managers’ responses to shock experiences.

## Uncertainty avoidance in international business

One common human challenge is that we do not know what the future might hold. Within this context, we act on deeply ingrained values. We know that these differ across cultures (Kluckhohn & Strodtbeck, 1961). But the extent to which we are tolerant about resultant unpredictability is highly variable (Venaik & Brewer, 2010). What we make of threats is not only relevant for individual dispositions but also for organisational processes and practice (Tayeb, 1995). Cross-cultural variations, then, might play a critical role in business decisions, too: When confronted with uncertain situations, which is particularly salient in the context of shocks, organisations might either act defensively (in order to minimise risks and damage) or aggressively (they understand shocks as a turning point and thus an occasion to do things differently) or rely on a combination of both options for action (Bundy et al., 2016). Crucially, while practising uncertainty is highly variable across cultures, Lee et al. (2020) have shown that the way people within one culture deal with the unknown is very stable.

As a consequence, a useful point of departure for exploring how Asian organisations and actors within them go about volatile and ambiguous landscapes is the Uncertainty Avoidance (UA) dimension of national culture across the most impactful conceptual models of national culture, that is, the one by Hofstede (1980), by Schwartz (1992) and the one based on the GLOBE study (House et al., 2004). UA, the extent to which a culture values predictability (Hofstede, 1980), is likely to augment the sense-making of shock events which we cannot cope with through standard routines. Those cultures high on UA think poorly of unstructured situations. Hence, they tend to either avoid such situations or try to quell uncertainties (and, connected to this, feelings of helplessness) as quickly as possible. The other extremes, low UA cultures, are marked by being more at ease with situations that are outside their control. Hence, they are less likely to mitigate uncertainty. In fact, they are okay with the possibility of continuous change, which they embrace flexibly and creatively (Parboteeah et al., 2005).

Building on these two extreme manifestations, we argue that it is not possible to understand responses to the unknown by merely applying rational considerations. Essentially, shock is a situation when we simply do not know what lies ahead. It might be a cousin of uncertainty (and, by extension, attempts to calculate risks), but its mishmash of surprise, rapidness and angst about losing control makes such events utterly different from reoccurring stressors (Ferretti et al., 2015). Cultural assumptions around the unknown, then, play a key role in guiding how we feel, think and act upon adversity.

To state the obvious: UA and risk behaviour are two separate domains (Hofstede, 2005). Indeed, it would be short-sighted to argue that those who are uncertainty avoidant act better or worse in a crisis (or vice versa). However, organisations and actors within them may experience exposure to significant adversity differently. Different experiences, then, are likely to result in different solutions to navigating a crisis, either biased defensively or biased experimenting. If culture matters in weathering latent dangers (Li et al., 2013), an exploration of UA amongst Asian members will provide context for what might make some organisations better able than others to bounce back or forward from turmoil.

A country’s culture is a key environmental determinant affecting both institutions and actors within them (Steenkamp et al., 1999; Triandis, 1989). Against this backdrop, UA creates social stimuli that either reinforce or punish responses to unknown situation contingent on dominant norms and beliefs in a country. Due to systematic UA differences, organisations in some countries value orderliness and consistency to reduce ambiguity, whereas in other countries, organisations might have less structure, processes and rules. Together, country differences in UA operate on what people think of uncertainty (cognitive), what emotions people associate with uncertainty (affective) and what people do about uncertainty (directive) (Usunier & Lee, 1999).

Because of their prominence in cross-cultural business research, we have chosen to look at the cultural values of the aforementioned three models. In Hofstede’s world, UA reflects the extent to which a society and its members appreciate, or feel uncomfortable with, uncertainty and ambiguity (Hofstede, 1980). Countries high on UA try to control ambiguous situations, whereas countries low on UA tend to let matters run their course. The GLOBE study defines UA as “the extent to which members of an organisation or a society strive to avoid uncertainty by reliance on social norms, rules and bureaucratic practices to alleviate the unpredictability of future events” (House et al., 2002, p. 5). High UA cultures rely on established social norms, rituals and bureaucratic practices to control an unpredictable future. Low UA cultures are more relaxed about ambiguities and less concerned with social rules. In the model of Schwartz (1992) and Schwartz and Boehnke (2004), the harmony dimension most closely resembles notions of uncertainty and how to deal with it (Imm Ng et al., 2007). High harmony members of a society emphasise accepting things as they are. Low harmony members tend to focus on how things could be changed for personal or group gains.

**Fig. 1:**
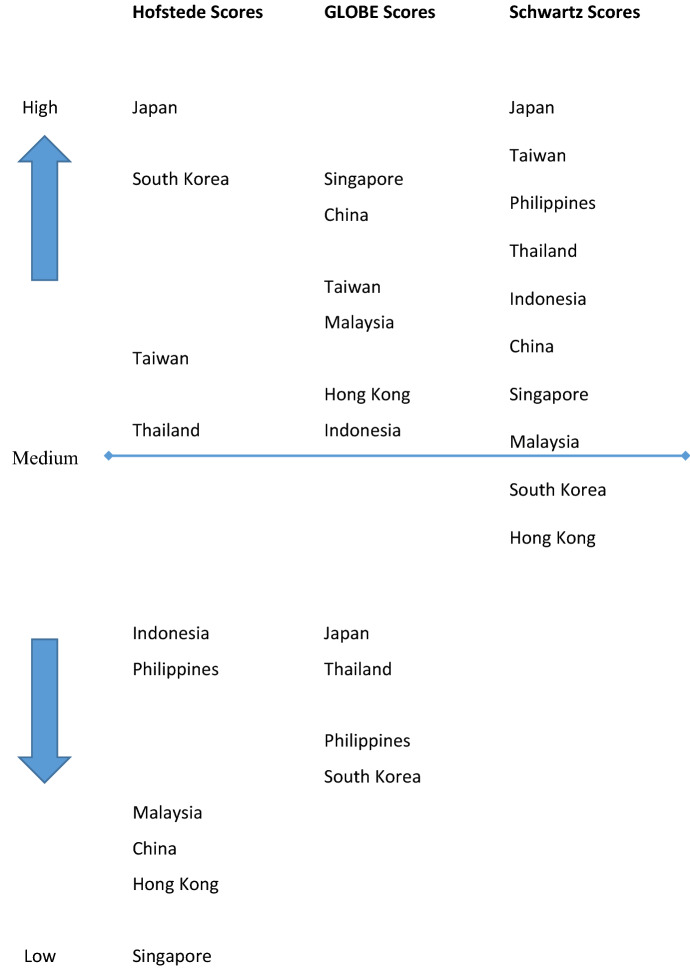
UA conceptualisations and scores for East and South-East Asian economies

All three models offer an understanding of how cultural assumptions infuse experiences associated with uncertainty and, by extension, how organisations and people within them move on from significant adversity. They do so using different UA definitions. In line with these divergent conceptualisation measurements, they, too, differ across Hofstede, GLOBE (social practises as they are, and how they should be) and Schwartz models (e.g. Alipour, 2019). This, in turn, might explain the substantial differences we find for the ten largest East and South-East Asian economies, measured in terms of GDP (Fig. 1). That being said, it is striking to see the differences between these countries generally and how sensitive these differences are to diverging specifications of UA, which still point in the same direction, after all.

In terms of geography, we detect substantial variations across East and South-East Asia. Asia is a continent full of dynamism but also full of contrasts when it comes to perceptions of uncertainty. The UA in-between country differences send out the clear warning that we should not lump all countries together. Instead, we should pay attention to divergent experiences of shock events across Asia and, by extension, likely divergences in dealing with them. In terms of the level of abstraction, there are also substantial variations. In this regard, we find the UA dimension for East and South-East Asia to have little overlap across the three conceptualisations. While central dispositions across members of all countries tend to accentuate a harmonious fit with things that cannot be changed, UA at the country and group level indicate quite contrary experiences (especially for Japan, South Korea, Singapore and China). Make no mistake: Beyond the much-discussed and admired legacy of stoicism in the face of significant adversity (Carteret, 2011), there seem to be strong cultural variations in tolerance for ambiguity and, by implication, restraint of action or venturesomeness. Organisations might belong to a certain country culture, but this does not mean they chime with country cultural experiences of uncertainty. Based on this, organisations and actors within them may act in ways distinctly different from country-level characteristics.

Finally, there also is a substantial temporal variation of UA among these countries. As you can see in Table 2, which is based on Taras et al. (2012) longitudinal meta-analytic review of UA based on Hofstede’s specification of this cultural dimension, many Asian countries show a volatile development of UA over the three decades captured by this data. Hence, there are not only marked differences between Asian countries and regions, but UA in these countries is also not carved in stone and further develops over time. This becomes notably clear for most East Asian countries, where we can observe a clear shift towards lower levels of UA.

**Table 2:** Uncertainty avoidance scores over time for selected Asian countries

Country	1980s	1990s	2000s
Japan	1.71	0.32	0.24
Taiwan	0.20	− 0.13	− 1.15
Philippines	0.21	0.30	0.11
Thailand	0.48	0.10	− 0.27
Indonesia	− 0.19	− 0.68	− 0.28
China	1.08	0.22	− 0.08
Singapore	− 1.86	− 0.54	− 1.19
Malaysia	− 0.69	0.34	− 0.64
South Korea	1.35	0.46	− 0.53
Hong Kong	− 0.34	− 0.53	− 0.10

Based on Taras et al. (2012)

All this suggests that UA is by no means a simple construct. As a starting point, there are variations of what actually makes a crisis and when and how it is perceived as such. What might be a catastrophic incident for some might be a mere hiccup for others. Past and present experiences build up UA and together shape attitudes towards the unknown. Take, for example, the notorious pessimism of Japanese CEOs about things that may happen (see, for instance, PwC surveys 2017). Such emphasis on undesirable outcomes might translate into better preparedness for unexpected events while, at the same time, foregoing opportunities (Tversky & Kahneman, 1974). By contrast, the confident outlooks of Chinese CEOs might obfuscate the accuracy of judgments, too. Such bias is likely to have a decisive influence on how turbulences in the external environment are being seen and the extent to which crises are being experienced. No doubt, substantial intra-country and inter-country variation across East and South-East Asian economies affects adaptive capabilities of organisations to act upon, adjust and recover from shock.

## Future research on the interplay of culture and shock responses

Realistically, leaders use a combination of logic and instinct (Simon, 1987), especially in the case of unpredictable events, when decisions have to be made in highly charged and emotional environments (Smith & Elliott, 2007). What is crucial is that organisations and their actors must give meaning to critical experiences when changes unfold in a disorderly manner (Weick, 1993). Evidently, views about uncertainty and, in turn, management thinking affect such sense-making processes. Whether and to what extent decisions move between rational and irrational considerations (Wright & Phillips, 1980) may therefore be less decisive than the ability of organisations to adapt to their environment through susceptibility to uncertainty, contextual thinking, and, perhaps, willingness to sit things out. What becomes clear through all these considerations is that research on the consequences of shocks for Asian businesses needs to take into account such contextual differences with regard to approaching uncertainty caused by shocks. Therefore, more research in more diverse Asian settings is required to capture this complexity.

In order to provide a framework, from which testable hypotheses can be systematically derived, could the way countries in Asia and Europe handle the coronavirus (Covid-19) crisis be a case in point? Overall, the starting position in the face of this tragic pandemic was not dissimilar for European and Asian regions. Factors such as urban development, closely knit and cross-border value chains, or regional tourism should have contributed to a rapid spread of infections in the same way. If anything, population density, social mobility or value chain spread and participation are much more pronounced in Asia than in Europe and, therefore, should have resulted in similar or even higher infections rates. It did not. In fact, the pandemic, which would run its course from Asia, occurred in Europe with some delay. Arguably, given this time advantage, uncertainties surrounding the virus’s social, health, or economic impact (and what measures best put in place) should have been higher in Asia than in Europe. Though we hasten to add that there are substantial variations across the Asian continent, we speculate that (based on expert interview data which we collected from May to June 2020 across Asia) that (i) strict rules and their quick enforcement, (ii) clarity of government communication and (iii) individual discipline and public willingness to give up on personal freedoms greatly helped control infection incidences. Europe and Asia also differed in their acceptance of contact tracing technology. In large parts of Asia, the widespread acceptance and use of technology (for instance, in Singapore or Hong Kong app-equipped smartphones) helped track and contain the virus. In our eyes, what set the two region’s responsiveness apart is Asia’s susceptibility to natural disasters (Bank, 2019) and the burden to pandemics (Peckham, 2016).

These surely have created a learning context that was activated swiftly and strictly at the first signs of a severe outbreak: In Wuhan, the Chinese government responded with monitoring, surveillance and preparation of medical facilities and supplies. In January, the Japanese government enforced quarantine measures (e.g. passengers of the cruise ship Diamond Princess were not allowed to leave) and entry restrictions. As seen in SARS (2002) and MERS (2015) outbreaks, Taiwan, too, has a history of dealing with infectious diseases. These experiences informed the cautiousness, care and determination with which the government (with broad public support) fought the coronavirus outbreak from the get-go. Note that all this happened in a period of high uncertainty (about the scale) and unpredictability (of mortality rates). European member states had time to prepare and put in place robust public measures. Yet, at the time, while the writing was on the wall for a historically unprecedented pandemic with far-reaching social and economic impact, efforts in Asia were widely characterised in Europe as Draconian. Instead of sound judgement based on trust in science (e.g. epidemiological modelling), Europe quarrelled over constitutional responsibility and saw the quick dismantling of supra-national organisations. Arguably, variations in UA interact with differences in reasoning references—with practical consequences for resolving shock: The European focus on universalist rules (“principles first!”) juxtaposes the Asian particularism and focus on interdependencies and results (“pragmatism-first”!) in dealing with the pandemic (Meyer, 2014; Trompenaars, 1996).

Shocks are intense and exhausting experiences. Organisations and actors within them all have some kind of response to a sudden and unexpected events. But they differ in the way they go about disruptive episodes prior, during and after a shock event. Although there is no simple relationship in dealing with a collapse of meaning between uncertainty assumptions and learning approaches, we collectively find that the two interact and are part of resultant differences in adaptation within the context of significant adversity. When we step back from our findings and those of the three featured articles in this Special Issue, it is notable that responses to shock trigger resourcefulness, learning and (business) transformation. But there are variations (i) in the way business actors make sense of ambiguous situations and, in turn, (ii) in the extent to which they keep going (no matter what is thrown at them). The relationship between these two culturally induced characteristics provides a rich context for resilience research and its practical application to Asian Business contexts.

Greater acceptance of the unknown appears to give prerequisite to approaching poorly structured situations in an intuitive manner. Such pragmatism, or particularism, allows for ongoing adjustment and correction of a set direction. By contrast, organisations that feel uncomfortable with uncertainty might see in non-routine events exceptions that weaken the “rule of the game.” Instead of emphasising the particular needs of a situation, the analysis (and perhaps isolation) of threat factors is in focus. Such principle lead, deductive reasoning, or universalism, might come at the cost of flexibility on the one hand and relatively quick implementation of response measures (once they are formulated) on the other. Table 3 maps the UA dimension (high versus low US) with Reasoning approaches (universalism versus particularism).

**Table 3:** Interplay of cultural characteristics and shock responses

		Universalism “Principles first” “Think things through”	Particularism “Pragmatism first” “Learning by doing”
Uncertainty avoidance	High	Unusual events as exceptions to rules & regulations Strong need to regain control Dogmatism and struggles over how to intervene	Little choice but endurance, especially for things outside one’s control Determination to learn Incremental adaptation
	Low	Awareness of limitations to pre-empt extreme events Shock sets precedent for new rules to become active Ride things out	Appreciation of permanent change Emphasis in creativity Receptiveness to innovation

There is little doubt that firms’ growth (and even survival) hinges on how effective they are in coping with non-routine landscapes. Our framework suggests that some have a better grip on radical uncertainty than others.

- Actors high in UA with universalist reasoning (upper left-hand quadrant) have a need for controlling all eventualities. They usually do so by hammering out rules and processes that seek to pre-empt helplessness before it occurs. Events that fall outside practised uncertainty frames shatter the perceived ability to control events. Struggles over how to adapt to the new normal and to avoid similar surprises in the future result from that.
- By contrast, particularist actors low in UA (lower right-hand quadrant) are comfortable with the unknown and are, thus, more likely to embrace change. For them, there is no “one best way” of practising uncertainty, and they do not take action in order to prevent all kinds of unforeseeable events from happening. Instead of thorough planning, they believe in coming up with solutions through hands-on learning. For them, shocks are positive as they induce innovation.
- For those actors high on UA with particularist reasoning (upper right-hand quadrant), ambiguity means discomfort. However, for them, the best response to fears of chaos and vulnerability (that might come with radical uncertainty) is not excessive control. Business landscapes are never permanent, and an essential determinant of managing disruptions is a commitment to humbleness and ongoing learning. Gradual adaptation increases control and creates step-by-step favourable conditions for future action.
- Universalist actors low in UA (lower left-hand quadrant) tolerate ambiguity. For them, economic and social costs associated with excessive response planning for extreme events are, on balance, high. Instead of anticipating all eventualities or developing complex response processes, they might be inclined to sit chaos out or move attention to implementing new rules if and when a shock makes adjustments necessary.

In addition to explaining intra-Asian differences and between them and the rest of the world, each quadrant should offer insights into how radical uncertainty is practised. Thus, this framework can be the starting point for future theorising on the interplay of culture and shock responses and to derive hypotheses for future empirical research on this important topic. Given the large scale and long duration of the still ongoing Covid-19 pandemic, there should be sufficient data for this purpose.

## Conclusion

In this editorial, we reviewed the business and management research on shocks in Asia, including the articles in this special issue, and we discussed the role of the uncertainty avoidance dimension of national culture in international business and further combine it with the universalism-particularism dimension to discuss future research directions. Altogether, the articles in this special issue contribute to a more profound understanding of how individuals or organisations respond to the non-routine environment and sudden shocks conceptually (Hoegl and Hartmann, 2020) and empirically in the Asian context (Aman et al., 2021; Ha, 2021; Meyer-Ohle, 2021). Moreover, meaningful synergies between the findings from the studies in this special issue already become apparent. In this regard, the three articles by Aman et al. (2021), Meyer-Ohle (2021) and Ha (2020) suggest that improving business models and imitation of successful peers are possible responses in non-routine environments and sudden shocks by considering risks and uncertainty. However, while the improvement of business models and ecosystems would strengthen the capability to bounce back from the setbacks and address the ground challenges as shown in the Japanese case by Meyer-Ohle (2021) and the Kazakhstan case by Aman et al. (2021), the Korean case by Ha (2020) shows imitation of successful peers may be less effective as a means of learning and development to strengthen competitiveness and resilience.

That being said, the collection of this special issue alone may not provide a comprehensive understanding of the complexity of the Asian context in terms of the business under the non-routine environment. Beyond the insights provided by the articles included in this special issue, we hope that it also serves as a catalyst to stimulate more and more diverse empirical research on the consequences of shocks for Asian businesses and triggers scholars to take this challenge and direct their attention to this timely and relevant research area. Thus, we call for more studies in this critical area in the future and proposed several worthwhile avenues for future research on shocks in the Asian business context.

## Footnotes

1 This special issue comprises a selection of extended versions of papers presented at the 36th Euro-Asia Management Studies (EAMSA) annual conference: Non-routine Environments: European-Asian Business in Times of Shock, held in Austria, 2–5 October 2019. We wish to extend our gratitude to the Editor-in-Chief and the conference and special issue reviewers for their encouraging comments and perseverant guidance.
